# Supplementary material for: Ultrasound Versus Computed Tomography for Diaphragmatic Thickness and Skeletal Muscle Index during Mechanical Ventilation
Source: Diagnostics (Basel). 2022 Nov 21;12(11):2890. doi: 10.3390/diagnostics12112890 (PMC9689333; doi:10.3390/diagnostics12112890)
Supplement: Supplementary file 1 [file diagnostics-12-02890-s001.zip › Supplemental Figure S2.pdf]

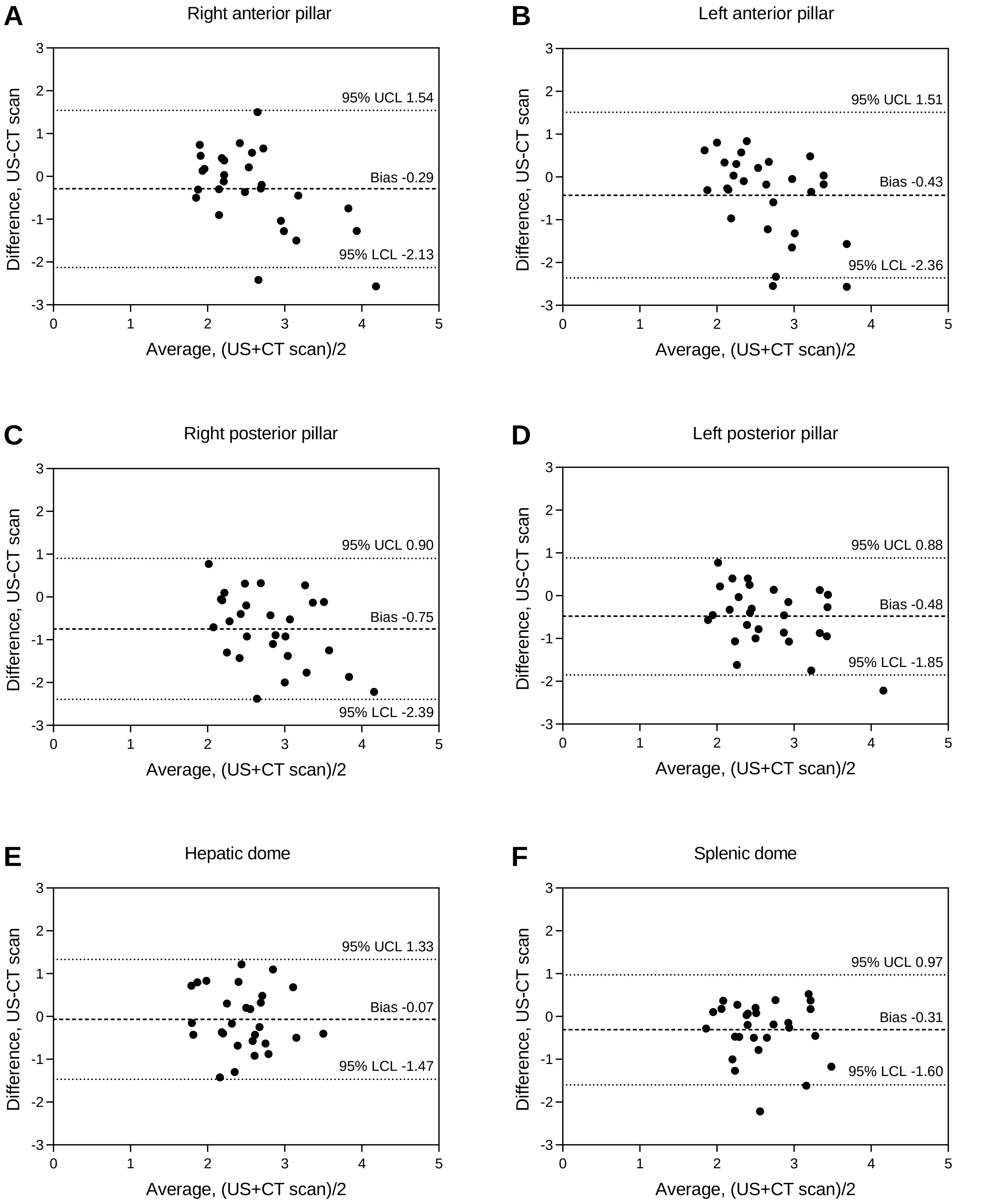
A
Right anterior pillar
95% UCL 1.54
Bias -0.29
95% LCL -2.13
B
Left anterior pillar
95% UCL 1.51
Bias -0.43
95% LCL -2.36
C
Right posterior pillar
95% UCL 0.90
Bias -0.75
95% LCL -2.39
D
Left posterior pillar
95% UCL 0.88
Bias -0.48
95% LCL -1.85
E
Hepatic dome
95% UCL 1.33
Bias -0.07
95% LCL -1.47
F
Splenic dome
95% UCL 0.97
Bias -0.31
95% LCL -1.60
Difference, US-CT scan
Average, (US+CT scan)/2
