# Supplementary material for: Ultrasound Versus Computed Tomography for Diaphragmatic Thickness and Skeletal Muscle Index during Mechanical Ventilation
Source: Diagnostics (Basel). 2022 Nov 21;12(11):2890. doi: 10.3390/diagnostics12112890 (PMC9689333; doi:10.3390/diagnostics12112890)
Supplement: Supplementary file 1 [file diagnostics-12-02890-s001.zip › Supplemental Table S1.pdf]

**Demographic and clinical features**	**N=29**
Age, years	55 ± 14
BMI, kg/m^2	24.7 (22.5 – 27.5)
Sex, n (%) male	22 (75.9%)
Enrolled patients, n (%):	
• Monza	20 (70%)
• Milano	9 (30%)
Controlled MV before enrollment, days	4 (2 – 8)
Assisted MV before enrollment, days	4 (1– 6)
Total ventilation before enrollment, days	9 (5 – 14)
Fraction of total time spent on assisted MV, %	44 ± 34
Neuromuscular blockers administered at any time, n (%)	22 (75.9%)
Days of neuromuscular blocking	3 (2 – 7)
High doses of corticosteroids* administered at any time, n (%)	9 (31%)
Comorbidity, n (%):	
• Hypertension	6 (21%)
• COPD	1 (3.4%)
• DM	3 (10.3%)
• Malignancy	5 (17.2%)
• CKD	1 (3.4%)
• Immunosuppression	7 (24.1%)
• Cardiac failure (NYHA class III – IV)	2 (6.9%)
• Liver failure (Child-Pugh C)	1 (3.4%)

Primary reason for ICU admission, n (%):	
• ARDS	11 (37.9%)
• Asthma	1 (3.5%)
• Other respiratory failure	6 (20.7%)
• Sepsis or septic shock	5 (17.2%)
• Trauma	3 (10.3%)
• Severe acute pancreatitis	2 (6.9 %)
• Liver failure	1 (3.5%)

Supplemental Table S1. Demographic and clinical characteristics of enrolled patients at the time of enrollment. Definition of abbreviations: ARDS = acute respiratory distress syndrome; BMI = body mass index; CKD = Chronic kidney disease; COPD = chronic obstructive pulmonary disease; CT-scan = Computerized Tomographic Scan; DM = Diabetes mellitus; ICU = intensive care unit; NYHA = New York Heart Association; SD = standard deviation; US = ultrasound. * High doses of corticosteroids: > 1 mg/kg of Methylprednisolone.
