# Supplementary material for: Ultrasound Versus Computed Tomography for Diaphragmatic Thickness and Skeletal Muscle Index during Mechanical Ventilation
Source: Diagnostics (Basel). 2022 Nov 21;12(11):2890. doi: 10.3390/diagnostics12112890 (PMC9689333; doi:10.3390/diagnostics12112890)
Supplement: Supplementary file 1 [file diagnostics-12-02890-s001.zip › Supplemental Table S2.pdf]

Supplemental Table S2. Correlation between Computerized Tomography measurements.

Diaphragmatic areas sampled by CT-scan		Right posterior pillar	Hepatic dome	Left anterior pillar	Left posterior pillar	Splenic dome
**Right anterior pillar**	r	0.604	0.754	0.644	0.531	0.638
	p-value	0.001	<0.001	<0.001	0.004	<0.001
**Right posterior pillar**	r		0.376	0.579	0.779	0.557
	p-value		0.049	0.001	<0.001	0.002
**Hepatic dome**	r		1	0.622	0.436	0.553
	p-value			<0.001	0.020	0.002
**Left anterior pillar**	r			1	0.603	0.709
	p-value				0.001	<0.001
**Left posterior pillar**	r				1	0.679
	p-value					<0.001
**Splenic dome**	r					1
	p-value					

Pearson correlation coefficient (r) and p-value between diaphragmatic thickness measurements using CT-scan. Definition of abbreviations: CT-scan = Computerized Tomographic Scan.
