# Supplementary material for: Ultrasound Versus Computed Tomography for Diaphragmatic Thickness and Skeletal Muscle Index during Mechanical Ventilation
Source: Diagnostics (Basel). 2022 Nov 21;12(11):2890. doi: 10.3390/diagnostics12112890 (PMC9689333; doi:10.3390/diagnostics12112890)
Supplement: Supplementary file 1 [file diagnostics-12-02890-s001.zip › Supplemental Table S3.pdf]

Supplemental Table S3. Correlation between CT measurements of the diaphragm and levels of skeletal muscle index.

Diaphragmatic areas Sampled by CT scan		Correlation with sarcopenia (SMI = TMA/h^2)
**Right anterior pillar**	r	0.354
	p-value	0.070
**Right posterior pillar**	R	0.055
	p-value	0.784
**Hepatic dome**	r	0.170
	p-value	0.395
**Left anterior pillar**	r	- 0.005
	p-value	0.993
**Left posterior pillar**	r	0.200
	p-value	0.317
**Splenic dome**	r	0.161
	p-value	0.423
**Mean diaphragmatic thickness**	r	0.194
	p-value	0.332

Pearson correlation coefficient (r) and p-value between diaphragmatic thickness measurements using CT-scan and skeletal muscle index. Definition of abbreviations: CT = computed tomography; h = height expressed as meter; SMI = skeletal muscle index; TMA = total muscle area.
