# Supplementary material for: Ultrasound Versus Computed Tomography for Diaphragmatic Thickness and Skeletal Muscle Index during Mechanical Ventilation
Source: Diagnostics (Basel). 2022 Nov 21;12(11):2890. doi: 10.3390/diagnostics12112890 (PMC9689333; doi:10.3390/diagnostics12112890)
Supplement: Supplementary file 1 [file diagnostics-12-02890-s001.zip › Supplemental Table S4.pdf]

Supplemental Table S4. Comparison of patients with Low versus high Skeletal Muscle Index (SMI).

**Variable**	**Low SMI (n=14)**	**High SMI (n=14)**	**p-value**
Age	61±13	50±13	0.028
BMI	24±3	27±5	0.181
Sex, M	10 (71)	11 (79)	0.663
Ventilation pre-enrollment, days	10 (4-18)	8 (6-13)	0.594
Control MV before enrollment, days	5 (3-14)	3 (2-6)	0.268
Control MV before enrollment, days	4 (0-7)	4 (2-6)	0.723
Fraction of total time spent on assisted MV, %	30 (0-63)	54 (33-78)	0.159
Patients on NMB, n (%)	11 (79)	10 (71)	0.663
Days on NMB, days	5 (3-13)	3 (0-4)	0.064
Monza<br>Milano	10 (71)<br>4 (29)	9 (64)<br>5 (36)	1.000
Steroids, n (%)	6 (43)	3 (21)	0.420
Comorbidity			
• Hypertension	5 (36)	1 (7)	0.165
• COPD	1 (7)	0 (0)	1.000
• DM	3 (21)	0 (0)	0.222
• Malignancy	4 (29)	1 (7)	0.326
• CKD	1 (7)	0 (0)	1.000
• Immunosuppression	5 (36)	2 (14)	0.385
• Cardiac failure	2 (14)	0 (0)	0.481
• Liver failure	0 (0)	1 (7)	1.000
ICU admission			
• ARDS	7 (50)	4 (29)	0.440
• Asthma	0 (0)	1 (7)	1.000
• Sepsis	2 (14)	3 (21)	1.000
• Other	4 (29)	2 (14)	0.648
• Trauma	0 (0)	2 (14)	0.481
• Pancreatitis	1 (7)	1 (7)	1.000
• Liver failure	0 (0)	1 (7)	1.000
Duration of MV, days	25 (16-49)	20 (15-36)	0.434
Right anterior pillar, mm	2.6±0.8	2.8±1.2	0.542
Left anterior pillar, mm	3.0±1.0	2.7±0.8	0.357
Right posterior pillar, mm	3.2±0.6	3.2±1.0	0.962
Left posterior pillar, mm	2.8±0.6	3.0±0.9	0.460
Hepatic dome, mm	2.5±0.5	2.4±0.6	0.641
Splenic dome, mm	2.8±0.6	2.8±0.6	0.963

Mean diaphragmatic thickness (by CT), mm	2.8±0.6	2.8±0.7	0.989
Diaphragmatic thickness (by US, mm	2.1±0.4	2.7±0.5	<0.001

Definition of abbreviations: ARDS = acute respiratory distress syndrome; BMI = body mass index; CKD = Chronic kidney disease; COPD = chronic obstructive pulmonary disease; DM = Diabetes mellitus; ICU = intensive care unit; NYHA = New York Heart Association; SD = standard deviation; SMI= Skeletal Muscle Index; US = ultrasound, CT-scan = Computerized Tomographic Scan. * High doses of corticosteroids: > 1 mg/kg of Methylprednisolone.
