# Supplementary figures and images for: Ultrasound Versus Computed Tomography for Diaphragmatic Thickness and Skeletal Muscle Index during Mechanical Ventilation
Source: Diagnostics (Basel). 2022 Nov 21;12(11):2890. doi: 10.3390/diagnostics12112890 (PMC9689333; doi:10.3390/diagnostics12112890)

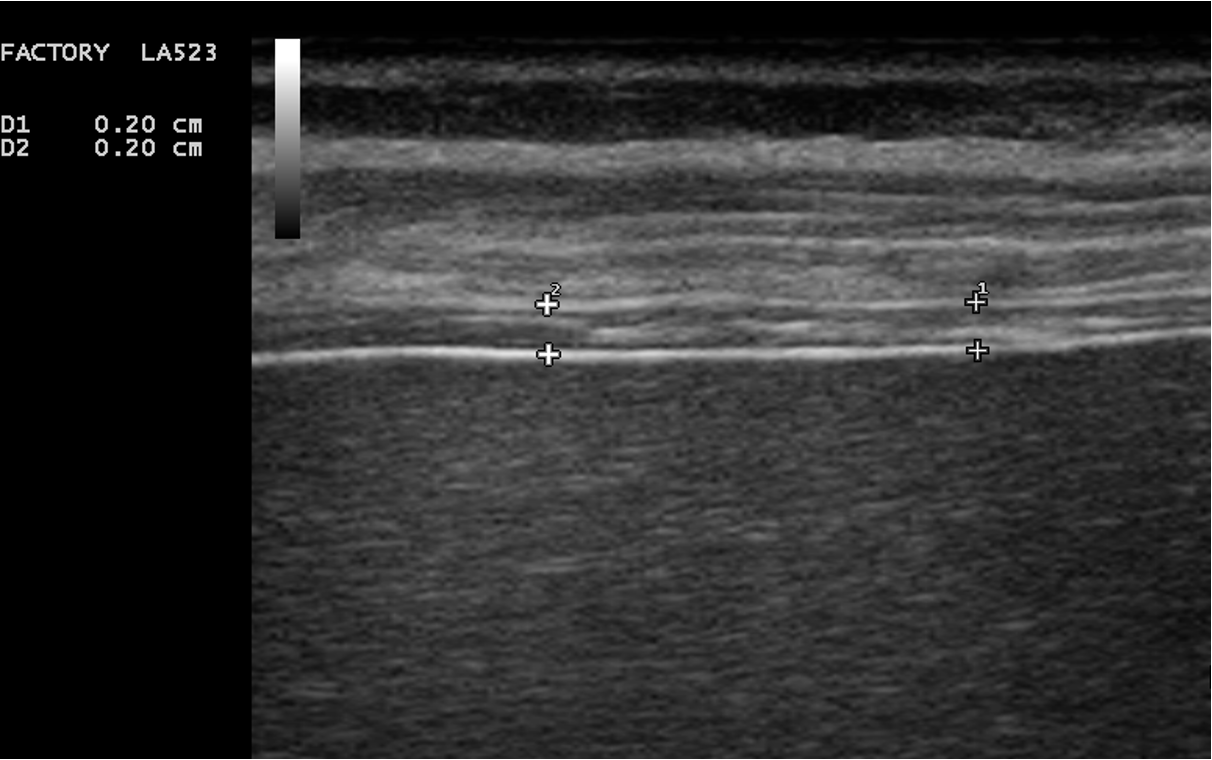

Supplement: Supplementary file 1 [file diagnostics-12-02890-s001.zip › Supplemental Figure S1.png]
